# Nutrition inequities in the Deaf community: a call for inclusive public health action

**DOI:** 10.1017/S1368980026101864

**Published:** 2026-01-23

**Authors:** Armineh Rajabi, Dilek Aslan

**Affiliations:** 1 Hacettepe University, Institute of Health Sciences, Department of Public Health (Nutrition and Food Sciences Program), Ankara, Türkiye; 2 Hacettepe University, Faculty of Medicine, Department of Public Health, Ankara, Türkiye

**Keywords:** Deaf nutrition, Nutrition inequities, Disability and public health, Hearing impairment and diet, Food insecurity in Deaf communities

## Abstract

Research shows that understanding the nutritional status and eating habits of people with disabilities is essential for creating effective preventive healthcare strategies. Communication barriers in healthcare settings, low health literacy and socioeconomic inequities are among the challenges this community faces. These difficulties can lead to poor nutritional knowledge, food insecurity and chronic diseases. Deaf individuals also experience higher rates of undernutrition, obesity and micronutrient deficiencies, primarily due to limited access to linguistically appropriate nutrition education. This commentary aims to explore the nutritional problems in the Deaf community, their determinants and risks and to offer inclusive solutions and preventive strategies at the individual, community and policy levels to ensure equitable nutrition and health for all Deaf individuals.

## What nutrition challenges do Deaf communities face?

Hearing loss is a growing global public health challenge. Current estimates suggest that over 1·5 billion people globally, representing nearly one-fifth of the world’s population, live with some level of hearing loss^(1)^. Among these individuals, approximately 430 million experience disabling hearing loss, and this number is estimated to exceed 700 million by 2050^(1)^.

Access to ear and hearing care services is still limited due to a global shortage of trained professionals and specialists^(1)^. This gap is more evident in low-and middle-income countries, where the need for healthcare services is highest. Consequently, fewer than one in five people who need hearing care services are able to receive them. Unaddressed hearing loss carries a substantial economic impact, with global costs estimated at nearly US$1 trillion annually^(1)^.

Nutrition plays an essential role in maintaining health and preventing disease. Although hearing loss is one of the most prevalent sensory impairments worldwide, nutrition-related challenges among individuals with hearing impairment are often neglected^(2)^. Despite the well-known and widely promoted importance of health literacy, many Deaf individuals face significant barriers to understanding medical terms and treatments, often due to a lack of sign language proficiency among healthcare professionals^(3)^.

Food insecurity is also a common concern among Deaf adults, which is associated with low income, depression and reduced quality of life^(4)^. According to the U.S. Household Food Security Survey screener, many adults with hearing impairment reported difficulty making food supplies last and lacked the financial means to purchase more within the past year^(5)^. Moreover, adults with hearing loss may experience a reduced ability to shop for and prepare food, alongside financial strain due to poorer physical health, consequently increasing the risk of food insecurity^(6)^.

Evidence also indicates that Deaf adults experience a higher burden of obesity, type 2 diabetes risk and CVD risk factors compared with hearing populations^(7)^. In a UK-based study of 298 Deaf British Sign Language users, obesity prevalence was high, particularly among older adults^(7)^. Rates of high blood pressure were higher than those reported in the Health Survey for England (37 % *v*. 21 %), with 29 % of participants unaware of their condition and 42 % having poorly controlled blood pressure despite having initiated treatment. In addition, one-third of participants had elevated total cholesterol levels. Although self-reported CVD was less prevalent than in the general population, treatment rates were substantially lower^(7)^.

According to another study in the USA, adults with hearing loss were more likely to report chronic diseases such as CVD, compared with adults with good hearing, and disparities increased as the severity of hearing loss worsened^(8)^. Adults with hearing loss reported lower alcohol consumption but were substantially more likely to be unable to engage in regular moderate or vigorous physical activity^(8)^. Moreover, a systematic review reported consistent evidence of health inequalities among Deaf signing populations compared with the general population, including a higher prevalence of obesity, hypertension and mental health problems such as depression and anxiety, as well as poorer quality of life and overall health status in this population^(9)^.

Beyond physical health, these challenges diminish the overall quality of life. The US Department of Agriculture has reported an association between disability, poverty and food insecurity, noting that disability substantially increases the risk of living in poverty, which in turn increases the chances of experiencing food insecurity, considering that individuals with disabilities also face higher healthcare costs^(2)^. In addition, those living with food insecurity are more susceptible to developing major depression or anxiety disorders^(4)^. Evidence shows that Deaf children also witness higher rates of undernutrition, stunting and dental problems in comparison with other children^(2)^.

## How can we prevent nutrition inequities in Deaf communities?

Closing the nutrition gap in Deaf communities requires a comprehensive, life-course approach. Many policies use one-size-fits-all approaches, lack disability-disaggregated data and exclude Deaf voices from decision-making. Primordial, primary, secondary and tertiary preventive strategies may mitigate the influence of the threat within the Deaf community. Examples are provided in Table 1.

**Table 1:** Prevention strategies to diminish nutritional inequalities in Deaf communities

Prevention level	Aim	Interventions
Primordial	Tackling root causes before they emerge	Inclusive education on sign language for healthcare professionals. Universal health coverage and legislation, promoting equity and inclusion for Deaf people. Social protection to reduce poverty, food insecurity and exposure to unsafe food.
Primary	Promoting healthy nutrition before the onset of problems	Developing nutrition education materials in sign language Provision of workshops and campaigns and integration of nutrition topics into school curricula Access to fortified foods and supplements, where needed Accessible food purchasing options Sign-language-supported guidance on grocery shopping (in person and online) Deaf-friendly basic cooking and meal-planning programmes Support for food label interpretation Peer support across different educational levels, including university education^(10)^
**Secondary**	Early detection and treatment	Screen for undernutrition, obesity, anaemia and dental issues at Deaf schools. Provide dietary consultations with interpreters for high-risk children and adults.
**Tertiary**	Minimising the impact of disability by preventing complications and improving quality of life through proper management and support	Rehabilitation for Deaf patients with chronic diseases. Ensuring nutrition monitoring and multidisciplinary care that includes dietitians, audiologists, interpreters and mental health professionals. Facilitating peer support groups to promote shared learning and motivation

Note. Prevention levels are based on the public health prevention framework, including primordial, primary, secondary and tertiary preventions.

## Examples of good practices

Governments and health organisations around the world have initiated implementing effective strategies to reduce health disparities and improve nutritional status among Deaf individuals. The WHO’s *International Classification of Functioning, Disability, and Health* provides a global framework for assessing health and disability at both individual and population levels^(2)^. As another example, the government of India has supported Deaf children by promoting their inclusion in mainstream schools to build confidence and encourage their participation in social activities^(2)^. The mid-day meal programme in India, which provides free nutritious lunches in public schools, has significantly reduced malnutrition and improved school attendance among children, especially in disadvantaged communities^(2)^. Targeted education programmes have also shown positive results in improving the nutritional status of Deaf people. For instance, a school-based programme that was implemented for 6 months for Syrian refugee children in Lebanon resulted in improvements in dietary knowledge, attitudes and BMI scores^(11)^.

## What will it take to close the nutrition gap for Deaf individuals?

Addressing persistent nutrition-related disparities among Deaf individuals requires a comprehensive, multi-level approach that integrates actions at the individual, community and policy levels (Table 2).

**Table 2:** A comprehensive approach for solving the problem

Intervention level	Details
Individual	Deaf individuals should have access to early, inclusive interventions such as sign-language-friendly nutrition education and visual learning materials. Improving health literacy and self-advocacy skills enables them to communicate confidently with healthcare providers and make informed food choices.
Community	Communities can bridge the gap by ensuring nutrition programmes reflect Deaf culture and communication needs. Schools and public health centres should use visual tools, plain and basic sign language. Training educators, health workers and service providers in Deaf cultural competence can create environments where Deaf individuals feel understood, respected and supported. Barriers such as difficulties in food purchasing and preparation need to be addressed by providing sign-language education for staff and retailers, guidance on navigating in-person and online grocery shopping, support for understanding food labels and visually or sign-language-supported cooking and meal-planning programmes.
Policy	At the policy level, stronger systemic change is needed to defend the rights of disabled communities. National health surveys (like National Health and Nutrition Examination Survey (NHANES) or National Family Health Survey (NFHS)) should include disability-related questions, specifically on hearing status, and ensure data are disaggregated by disability type. Deaf individuals should have representation in parliaments and policymaking bodies to protect their rights. Support programmes such as state disability pensions, along with services such as Integrated Child Development Services (ICDS) and Anganwadi centres, can help reduce undernutrition and improve the nutritional health among Deaf adults^(2)^.

Note. This table outlines a conceptual, multi-level public health framework (individual, community and policy) for addressing nutrition inequities in Deaf communities.

## Conclusion

Deaf communities face significant nutrition-related challenges that must be addressed. Closing this gap requires coordinated action across multiple levels. Ensuring accessible nutrition education, strengthening cultural and linguistic competence within health systems and integrating disability-disaggregated data into national surveys are some of the essential steps. Additionally, Deaf individuals should be actively involved in the development and evaluation of programmes and policies intended to support them. Implementing these strategies can help reduce the often ‘hidden’ burden of nutritional inequities experienced by Deaf individuals.

## Data Availability

This commentary does not contain original research data. No new datasets were generated or analysed for this work.
